# Mapping a Decade of Research: Bibliometric Analysis of Iron Overload in Chronic Kidney Disease

**DOI:** 10.7759/cureus.75766

**Published:** 2024-12-15

**Authors:** Abdulqadir J Nashwan, Jibin Kunjavara, Abdulrahman Al-Mashdali, Mohamed A Yassin

**Affiliations:** 1 Nursing & Midwifery Research Department, Hamad Medical Corporation, Doha, QAT; 2 Department of Oncology, Hematology and Bone Marrow Transplantation (BMT) Section, Hamad Medical Corporation, Doha, QAT; 3 Department of Hematology, Hamad Medical Corporation, Doha, QAT

**Keywords:** bibliometric analysis, chronic kidney diseases, hemochromatosis, iron overload, scientific mapping

## Abstract

This study conducts a bibliometric analysis (BA) to map the research landscape surrounding chronic kidney disease (CKD) and iron overload over the past decade. Utilizing PubMed as the primary database, a systematic search strategy was developed using BA guidelines, incorporating keyword and MeSH term refinements for comprehensive data retrieval. A Boolean operator-based search strategy was applied, capturing literature from 2014 to the first quarter of 2024, with inclusion criteria focusing on articles and review articles published in English. An initial pool of 408 records was narrowed to 52 articles after applying filters and exclusions based on relevance and document type. Data analysis employed the Java-based bibliometric tool VOSviewer, facilitating citation analysis, keyword co-occurrence mapping, and network visualization of author and institutional contributions. MeSH terms and keywords were analyzed for frequency and signal strength, with “Humans,” “Iron,” and “Iron Overload” emerging as dominant terms. Trends in publication activity from 2014 to 2024 revealed cyclic patterns influenced by external factors. The institutional analysis highlighted contributions from key healthcare and research institutions globally. The findings underline significant themes, contributors, and research gaps within the field, providing valuable insights for future CKD and iron management studies. This study not only identifies major trends and collaborative networks but also highlights potential areas for advancing patient care and treatment strategies for CKD patients with iron-related conditions.

## Introduction and background

Chronic kidney disease (CKD) is a progressive disorder that impairs kidney function and is often accompanied by complications, such as anemia and iron overload, which exacerbate patient outcomes. Approximately 850 million people worldwide are estimated to have kidney disease, most of whom live in low-income and lower-middle-income countries (LICs and LMICs), and a large proportion of these individuals lack access to kidney disease diagnosis, prevention, or treatment [1]. By 2040, CKD is projected to be the 5th highest cause of years of life lost (YLL) globally [2]. The prevalence of CKD has been reported to be higher in females than in males. In the United States, the age-adjusted prevalence of CKD stages 1-4 from 2015 to 2016 was 14.9% in females and 12.3% in males, nine similar to the sex-based differences reported in the global studies mentioned above [3]. A meta-analysis of 30 studies analyzing sex-stratified data found that CKD progresses more rapidly in men than in women. However, other research has suggested that nonbiological factors might influence this difference, including lifestyle, cultural, and socioeconomic elements. To better understand how sex impacts CKD incidence, prevalence, and progression, further investigation is needed, particularly in exploring potential sex-specific disease markers [4].

The reasons for these differences are unclear and are likely to be complex. Iron metabolism in CKD patients is of paramount concern, as impaired iron regulation can lead to either deficiency or toxicity, complicating disease management and treatment strategies [5]. Iron overload, frequently seen in patients undergoing hemodialysis or receiving erythropoietin-stimulating agents, poses significant health risks, including cardiovascular complications and increased oxidative stress [6].

Bibliometric analysis (BA) is increasingly used to map various healthcare topics’ scientific landscape. This method helps quantify research trends, collaboration patterns, and influential contributors to a field, offering insights into knowledge gaps and future research opportunities [7]. The present study conducts a BA of iron overload in CKD to elucidate emerging themes, leading contributors, and institutional involvement over the past decade. By identifying the research trends, this study aims to offer a comprehensive overview of the developments in the field, which can inform evidence-based interventions and foster collaboration among stakeholders in nephrology and iron management.

## Review

Methods

Data Sources

**Figure 1:**
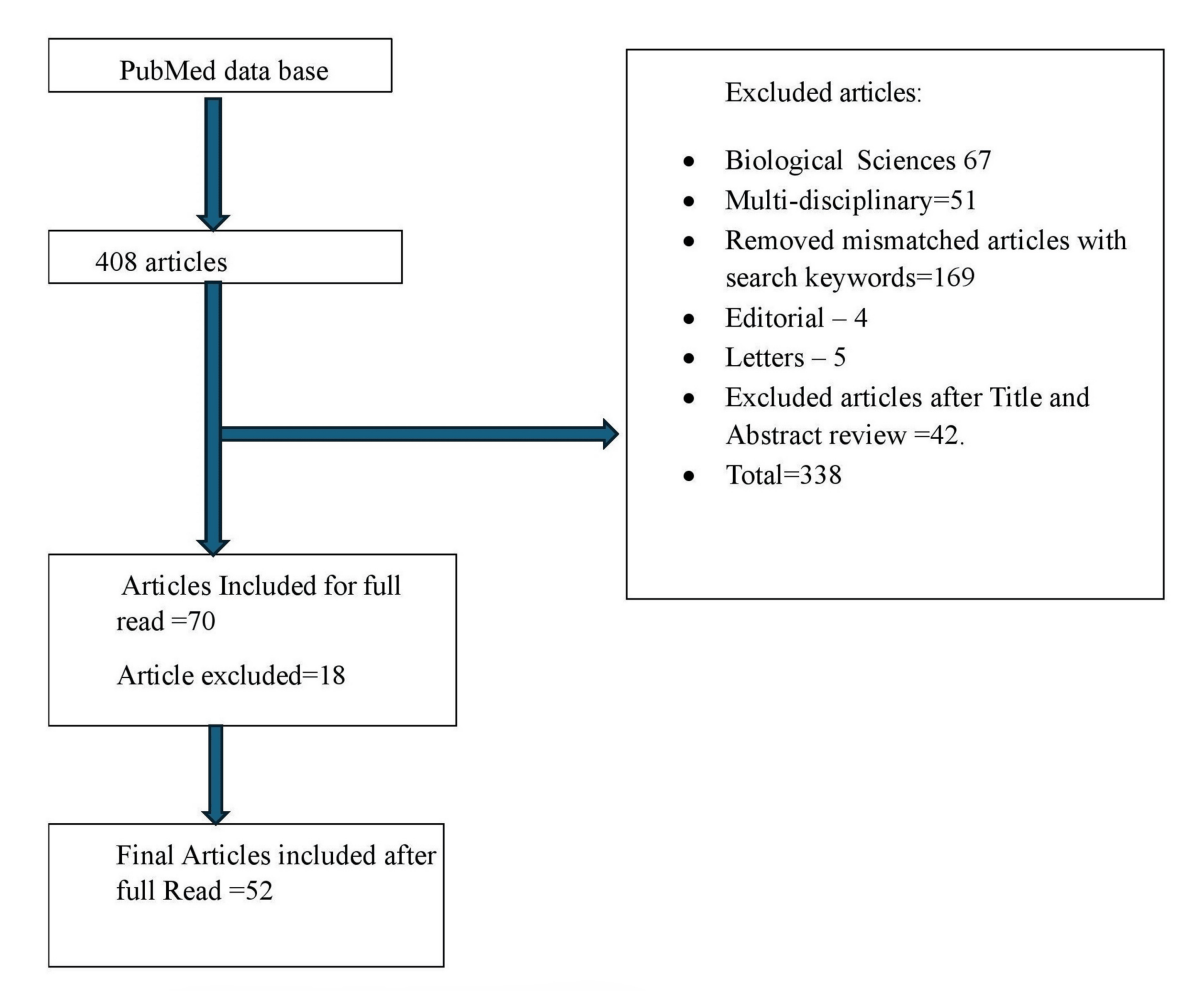
Flowchart of Article extraction for Bibliometric Analysis. The flowchart was created by the authors of the article.

This study adhered to the guidelines and steps outlined in a foundational article on BA. Common research databases include Web of Science, Dimensions, PubMed, Google Scholar, and Scopus, as these platforms allow correlation analysis across citation counts and article counts. However, to avoid biases related to varying bibliometric formats, PubMed was chosen as the sole database for the literature search. Based on BA guidelines, a systematic search strategy was developed, incorporating a literature review to refine keywords and select MeSH terms. Like Chronic Kidney Disease OR CKD OR Renal Failure (Chronic) OR Kidney Disease AND (Iron Overload OR Iron Excess OR Ferritin Overload) Filters: from 2014 - 2024. The Boolean operator was employed to focus on publications covering all these fields. An asterisk (*) was applied to capture various word endings. The search covered literature published in English from 2014 to the first quarter of 2024, including only articles and review articles. (Figure 1) outlines the data extraction procedure. The authors initially identified 408 records in PubMed using the specified search terms. After excluding non-article documents, items unrelated to health and health sciences, and 105 non-English publications, 52 records were retained for analysis.

Data analysis

The extracted data was analyzed using the Java-based bibliometric tool VOSviewer (version 1.6.18). VOSviewer is a tool for creating and visualizing bibliometric networks, such as co-authorship, co-citation, and keyword co-occurrence, allowing researchers to gain insights into trends, relationships, and the structure of academic literature. Total link strength, automatically calculated by VOSviewer and confirmed with Microsoft Excel, indicates the strength of collaboration, with thicker lines between nodes signifying higher levels of cooperation between items.

The bibliometric tools included network analysis to visualize co-authorship patterns and co-occurrence networks of MeSH terms and keywords. Frequency tables were constructed to display the number of occurrences of each MeSH term and author keyword. Signal strength metrics were calculated to assess the terms’ impact on the research landscape. Collaboration patterns among institutions were mapped to identify major contributors to the field. The analysis was complemented by visual tools, including density maps and line graphs, to illustrate trends and collaborative networks.

Findings

This study presents a comprehensive BA of research trends in iron burden related to CKD over the past decade. By mapping the scientific landscape, the study highlights key areas of focus, influential contributors, and region and country involvement in this field. The analysis is based on MeSH term co-occurrence, author and institutional contributions, keyword usage, and collaboration patterns among researchers and organizations.

The results provide valuable insights into the most frequently studied topics, the main contributors to research, and the emerging themes in CKD and iron overload research. By examining these patterns, the study helps identify knowledge gaps and potential areas for further investigation, with implications for enhancing patient care and treatment strategies related to iron management in CKD.

Trend in Publications on Chronic Kidney Disease and Iron Overload (2014-2024)

**Figure 2:**
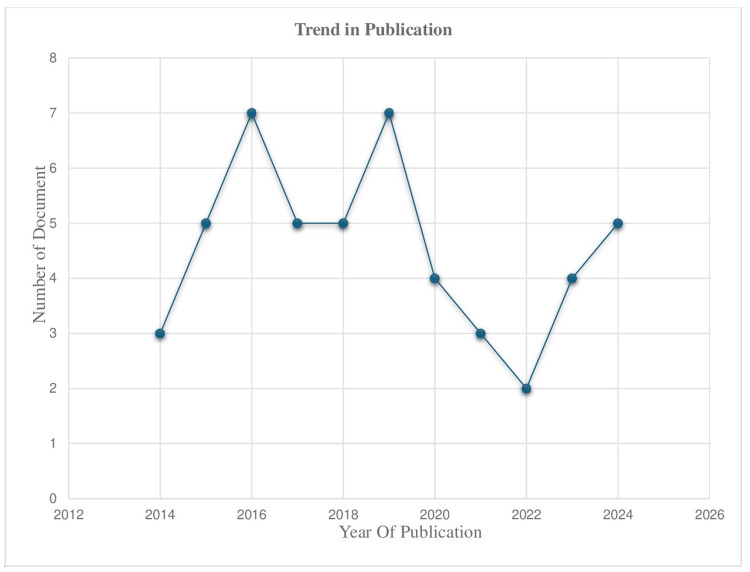
Publication Trend in the Years from 2014 to 2024. The image was created by the authors of the article.

The provided line chart below (Figure 2) shows the fluctuation in the number of documents published from 2014 to 2024. The trend demonstrates cyclical patterns, with peaks in publication activity occurring around 2015 and 2020, followed by notable declines, particularly in 2021 and 2022. After hitting a low point in 2022, the publication output shows signs of recovery in 2023 and 2024. The variability in publication trends suggests that external factors, such as funding availability or global events, may have influenced research efforts over the years.

Bibliometric Analysis of Co-occurrence and MESH Terms

The co-occurrence analysis of MeSH terms revealed the most frequently studied topics about CKD and iron overload. As shown in Table 1, the most common MeSH term was "Humans", appearing 32 times with a total signal strength of 160. This was followed by "Iron" (22 occurrences, total signal strength: 120) and "Iron Overload" (18 occurrences, total signal strength: 119), underscoring the focus on iron metabolism in CKD research. Terms such as "Renal Dialysis" (11 occurrences, signal strength: 81), "Ferritins" (10 occurrences, signal strength: 70), and "Hematinics" (11 occurrences, signal strength: 61) were also frequently used.

**Table 1:** Bibliometric Analysis of Co-occurrence and MeSH Terms.

S. No:	MeSH Terms	Occurrences (Nos.)	Total Signal Strength (Nos.)
1	Humans	32	160
2	Iron	22	120
3	Iron overload	18	119
4	Renal dialysis	11	81
5	Hematinics	11	61
6	Ferritins	10	70
7	Male	9	64
8	Anemia, iron deficiency	9	47
9	Kidney failure	6	37
10	Erythropoietin	5	27

**Figure 3:**
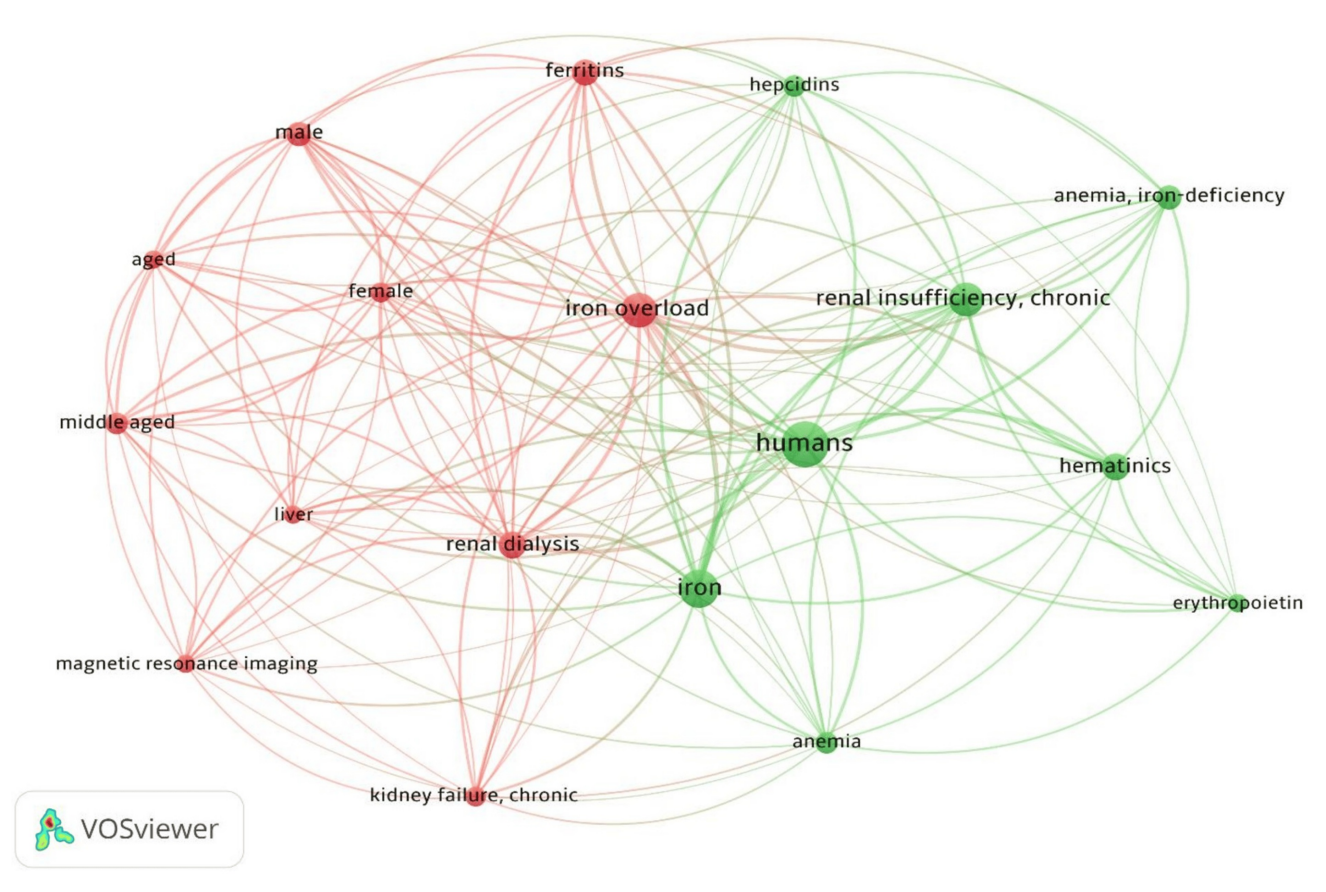
Co-occurrence Network of MeSH Terms Related to Chronic Kidney Disease and Iron Overload. The image was created by the authors of the article.

The MeSH terms "Kidney Failure" and "Erythropoietin" appeared less regularly, with 6 and 5 occurrences, respectively, suggesting the relative focus on other aspects of iron management in CKD. Illustrates the network of co-occurring MeSH terms, showing the interrelations among key research concepts (Figure 3).

Bibliometric Analysis of Co-occurrence and Authors Keywords

The analysis of author keywords provides insight into the central themes of CKD and iron overload research. As seen in Table 2, "Iron Overload" was the most frequently occurring keyword, with 18 mentions and a total signal strength of 24. Other prominent keywords include "chronic kidney disease" (16 occurrences, total signal strength: 23), "Anemia" (8 occurrences, signal strength: 18), and "Iron" (8 occurrences, signal strength: 14). Terms such as "Iron Deficiency", "Hemodialysis", and "Ferritin" appeared less frequently but still contributed significantly to the research landscape.

**Table 2:** Bibliometric Analysis of Co-occurrence and Authors Keywords.

S. No:	Keywords	Occurrences (Nos.)	Total Signal Strength (Nos.)
1	Iron overload	18	24
2	Chronic kidney disease	16	23
3	Anemia	8	18
4	Iron	8	14
5	Iron deficiency	5	11
6	Hemodialysis	5	9
7	Ferritin	5	7

**Figure 4:**
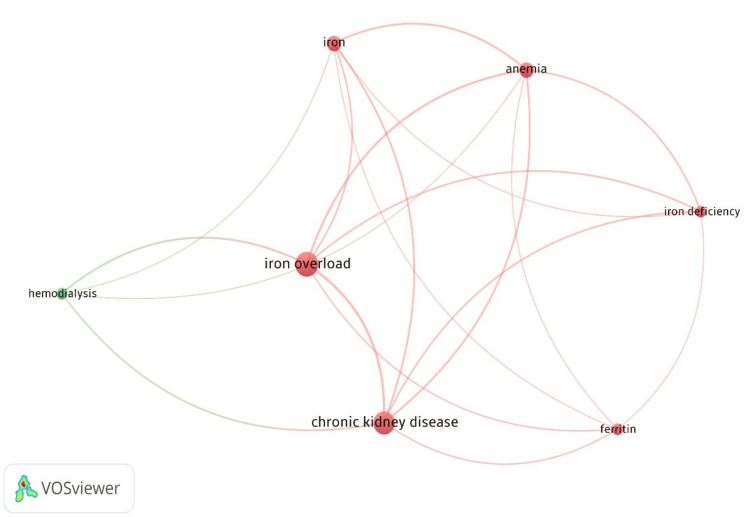
Co-occurrence Network of Key Terms in Chronic Kidney Disease and Iron Overload Research. The image was created by the authors of the article.

Figure 4 visualizes the co-occurrence of these key terms, providing a detailed map of the research trends in CKD and iron overload.

Bibliometric Analysis by Country and Region

The analysis of contributions to CKD and iron overload research reveals notable regional and country-specific participation, underscoring the global focus on this field. In the Middle East, Qatar, and other countries have shown significant involvement, reflecting regional health priorities driven by a high prevalence of CKD. The emphasis in this region is likely on understanding the public health impact and developing effective management and treatment strategies for iron-related complications in CKD patients. In Europe, contributions from Italy indicate a strong interest in CKD research, particularly relevant given the continent’s aging population and rising rates of kidney disease. European research generally focuses on nephrology, dialysis, and broader clinical management of CKD and iron overload.

This geographical diversity in contributions highlights the global response to CKD and iron-related issues, with specific areas of focus varying by region. The collaborative patterns across these regions emphasize the importance of international cooperation in addressing CKD’s complex health challenges and improving patient outcomes worldwide.

Discussion

Iron overload in CKD represents a significant clinical challenge that has garnered increasing attention in nephrology research over the past decade. This comprehensive BA examines the evolving landscape of scientific literature concerning iron overload in CKD from 2014 to 2024, encompassing 52 publications indexed in major databases. Through systematic evaluation of publication patterns, research themes, and collaboration networks, we present a detailed assessment of current knowledge, identify emerging trends, and highlight critical gaps in understanding. This analysis provides valuable insights into the trajectory of research efforts and suggests future directions for investigation in this crucial area of nephrology.

Iron is an essential element in human cellular physiology. It is incorporated into various proteins, including hemoglobin, catalases, lipoxygenases, ribonucleotide reductase, and cytochromes, facilitating a wide range of biochemical processes such as oxygen transport, energy production, DNA synthesis, immune defense, gene regulation, steroid and hormone synthesis, and drug metabolism [8]. Its versatility arises from its ability to participate in oxidation-reduction reactions by transitioning between ferric (Fe³⁺) and ferrous (Fe²⁺) states. This chemical property that makes iron crucial for cell growth and survival enables ferrous iron to react with peroxides, forming harmful hydroxyl or lipid radicals. Iron homeostasis is tightly regulated at the cellular, tissue, and systemic levels to maintain appropriate iron levels throughout the body [9]. Understanding these fundamental aspects of iron biology is crucial for appreciating the complex challenges in managing iron homeostasis in CKD patients.

Iron deficiency anemia is a significant complication in patients with CKD, leading most researchers to focus on monitoring anemia rather than the potential for iron overload resulting from iatrogenic iron supplementation [10]. Clinicians commonly use serum ferritin levels to assess iron status in CKD patients, as ferritin is a key indicator of the body’s iron stores. However, it is essential to note that various factors beyond iron levels can influence serum ferritin levels [11]. For instance, liver disease can significantly alter ferritin production and metabolism, leading to elevated serum ferritin levels even in normal or low iron stores. Inflammation is another critical factor; ferritin is often released during inflammatory states as part of the acute phase response, resulting in misleadingly high ferritin levels. This means that elevated serum ferritin may not accurately reflect iron overload and can complicate the interpretation of iron status in patients with conditions such as CKD. Other factors, such as infections, malignancies, and certain chronic diseases, can also affect ferritin levels, making it essential for clinicians to consider these variables when evaluating a patient’s iron status and determining the appropriate management strategies [12].

Patients with CKD may experience the consequences of iron overload, which can impact major iron storage sites. In the liver, excess iron can cause severe organ damage and ultimately lead to liver failure [4]. Cardiac iron overload is associated with arrhythmias and heart failure [4]. In contrast, iron accumulation in endocrine organs (such as the thyroid, pancreas, and pituitary gland) can result in dysfunction and delayed puberty in children [13]. Notably, the quantification of liver and cardiac iron and their relationship to serum ferritin levels have not been extensively studied in CKD patients compared to those with hemoglobinopathies. Consequently, there is no established cutoff value for ferritin in current clinical guidelines, highlighting the need for more reliable diagnostics for iron overload [14]. Given these serious clinical implications, developing effective management strategies has become a critical focus in nephrology research.

Managing iron overload in CKD patients is a complex and evolving area of research, with limited data available on effective strategies. This gap is primarily due to the variability in patient populations. CKD encompasses individuals with differing kidney functions, comorbidities, and treatment regimens, making it challenging to establish standardized protocols. Additionally, inflammation in CKD patients complicates the interpretation of iron status markers, such as serum ferritin and transferrin saturation, which can obscure iron overload assessment and management strategies effectiveness. Furthermore, there is a scarcity of large-scale, randomized controlled trials specifically focused on iron overload, with most existing studies concentrating on anemia management instead [15].

Additionally, the use of iron chelation therapy, such as deferasirox (DFX), in patients with CKD has generated controversy due to concerns about its safety and effectiveness. While iron chelation therapy is known to reduce iron overload in CKD patients effectively, its overall impact on this population is not fully understood. Some studies suggest that iron chelation therapy may increase the risk of kidney damage, whereas others report no significant adverse effects on kidney function. Additionally, there are concerns about how it might interact with other medications commonly prescribed to CKD patients. The appropriate dosing is also debated, with some research indicating that lower doses may be safer and more effective than higher doses. Overall, more research is needed to clarify these issues and guide the use of iron chelation therapy in CKD patients [16].

Our BA provides compelling evidence regarding the trajectory of iron overload research in CKD, revealing several key trends. The predominant focus on human studies, as indicated by the high frequency of "Humans" as a MeSH term (32 occurrences), aligns with recent meta-analyses highlighting the shift toward patient-centered research [17]. This transition from predominantly preclinical studies reflects the field’s maturation and responds to calls for more translational evidence, as emphasized in the KDIGO 2012 guidelines and their subsequent updates [3]. The strong co-occurrence of iron-related terminology ("Iron": 22 occurrences; "Iron Overload": 18 occurrences) parallels the growing recognition of iron homeostasis complexity in CKD, consistent with findings from large-scale studies such as the FIND-CKD trial [18]. The therapeutic focus is evidenced by the substantial representation of "Renal Dialysis" (11 occurrences) and "Ferritins" (10 occurrences), reflecting current clinical practice guidelines' emphasis on monitoring parameters. This pattern aligns with studies demonstrating the critical importance of precise iron status assessment in dialysis patients [19]. The concurrent prominence of "Hematinics" (11 occurrences) and "Erythropoietin" (5 occurrences) in the literature corresponds with contemporary approaches to anemia management, as validated by landmark trials such as PIVOTAL and FIND-CKD [20]. The emergence of "Anemia" as a frequently occurring keyword (8 occurrences) underscores its central role in iron homeostasis disturbances in CKD. This finding is particularly relevant given recent evidence from large cohort studies demonstrating the intricate relationships between iron deficiency, anemia, and adverse outcomes in CKD patients [21,22]. The network analysis reveals complex interconnections between these concepts, supporting an integrated approach to understanding and managing iron-related complications in CKD, as current clinical practice guidelines advocate.

The temporal analysis of publication patterns (2014-2024) reveals distinct fluctuations characterized by significant peaks in 2015 and 2020, followed by a marked decline from 2021 to 2022. The 2015 surge coincided with pivotal updates in clinical practice guidelines for iron management in CKD, while the 2020 peak represented the culmination of multiple large-scale clinical investigations [23]. The subsequent decline during 2021-2022 reflects the unprecedented impact of the SARS-CoV-2 pandemic on clinical research infrastructure and implementation [24]. The recovery trajectory in 2023-2024 demonstrates remarkable resilience within the research community and adaptation to post-pandemic research methodologies. This period has been characterized by renewed emphasis on chronic disease management and the strategic resumption of previously suspended research initiatives, indicating a positive trajectory for future investigations in this field.

Research gaps and future directions

Our BA has identified critical knowledge gaps requiring systematic investigation. A particularly significant deficit exists in understanding the mechanistic relationships between iron overload and cardiovascular complications in CKD patients. Despite cardiovascular disease representing the primary cause of mortality in this population, current publication patterns indicate insufficient exploration of this crucial association.

The contemporary research landscape reveals limited advancement in developing and validating novel biomarkers beyond conventional parameters such as ferritin and transferrin saturation. This represents a significant opportunity to expand the diagnostic armamentarium and enhance monitoring strategies for iron overload in CKD patients. Furthermore, our analysis identifies substantial gaps in understanding specific patient subgroups, particularly the pre-dialysis population and the influence of genetic determinants on iron metabolism disorders.

These identified research gaps necessitate comprehensive investigation through well-designed clinical studies addressing these underexplored aspects of iron overload in CKD. Future research initiatives should prioritize these areas to advance our understanding and optimize patient outcomes in this complex therapeutic domain. Developing innovative methodologies and integrating advanced technologies could address these knowledge gaps and improve clinical outcomes in CKD patients with iron overload.

Limitations

This study has several significant limitations that warrant consideration. The analysis was restricted to articles indexed with specific MeSH terms, potentially excluding relevant studies using different terminology, particularly in emerging research areas where standardized terminology is still evolving. The data collection's temporal limitation to the past decade may have missed valuable historical contributions and long-term trends. The reliance on bibliometric information introduces multiple biases, as metrics like signal strength and citation counts may only partially capture research impact, failing to account for practical applications, clinical relevance, or policy influence. Publication dynamics, including delays between research completion and publication and bias toward positive results, could affect trend analysis accuracy. Furthermore, the study could only partially account for external influences such as funding cycles, global events, regional differences in research capacity, institutional policies, technological advancements, and changes in publishing practices, all of which can significantly impact research output and direction but are challenging to quantify within a BA framework. These limitations suggest that while the study provides valuable insights, its findings should be interpreted within these methodological constraints.

## Conclusions

This decade-long BA provides comprehensive insights into the evolving landscape of iron burden research in CKD. Our findings reveal a dynamic research field centered on iron metabolism, particularly emphasizing iron overload, renal dialysis, and ferritin dynamics. The analysis identified significant publication patterns, showing both peaks and troughs over the studied period, with a notable resurgence in research activity during 2023-2024, suggesting renewed scientific interest. The analysis uncovered extensive collaborative networks among institutions and researchers, highlighting the global effort to address this critical aspect of CKD management. While substantial progress has been made in understanding iron homeostasis in CKD, our analysis also revealed critical knowledge gaps, particularly in novel therapeutic approaches and personalized iron management strategies. Despite external challenges, sustained research on the iron burden in CKD underscores its fundamental importance in nephrology. These findings provide a strategic framework for future research directions and emphasize the potential for translating scientific advances into improved clinical outcomes. Addressing the identified research gaps while building upon existing knowledge will be crucial for developing more effective, personalized iron management strategies, ultimately enhancing the quality of life for CKD patients. This analysis serves as a comprehensive review of past achievements and a roadmap for future investigations in this vital field of nephrology.

## References

[1] Jager KJ, Kovesdy C, Langham R, Rosenberg M, Jha V, Zoccali C (2019). A single number for advocacy and communication—worldwide more than 850 million individuals have kidney diseases. Kidney Int.

[2] Grams ME, Rebholz CM, McMahon B (2014). Identification of incident CKD stage 3 in research studies. Am J Kidney Dis.

[3] Hill NR, Fatoba ST, Oke JL, Hirst JA, O'Callaghan CA, Lasserson DS, Hobbs FD (2016). Global prevalence of chronic kidney disease—a systematic review and meta-analysis. PLoS One.

[4] Bairey Merz CN, Dember LM, Ingelfinger JR (2019). Sex and the kidneys: Current understanding and research opportunities. Nat Rev Nephrol.

[5] Drüeke TB (2000). Hyperparathyroidism in Chronic Kidney Disease. Endotext [Internet].

[6] Pergola PE, Fishbane S, Ganz T (2019). Novel oral iron therapies for iron deficiency anemia in chronic kidney disease. Adv Chronic Kidney Dis.

[7] Donthu N, Kumar S, Mukherjee D, Pandey N, Lim WM (2021). How to conduct a bibliometric analysis: An overview and guidelines. J Bus Res.

[8] Yiannikourides A, Latunde-Dada GO (2019). A short review of iron metabolism and pathophysiology of iron disorders. Medicines.

[9] Hentze MW, Muckenthaler MU, Andrews NC (2004). Balancing acts: Molecular control of mammalian iron metabolism. Cell.

[10] Macdougall IC, Bircher AJ, Eckardt KU (2016). Iron management in chronic kidney disease: Conclusions from a "Kidney Disease: Improving Global Outcomes" (KDIGO) Controversies Conference. Kidney Int.

[11] Kalantar-Zadeh K, Kalantar-Zadeh K, Lee GH (2006). The fascinating but deceptive ferritin: To measure it or not to measure it in chronic kidney disease?. Clin J Am Soc Nephrol.

[12] Dignass A, Farrag K, Stein J (2018). Limitations of serum ferritin in diagnosing iron deficiency in inflammatory conditions. Int J Chronic Dis.

[13] Gattermann N, Muckenthaler MU, Kulozik AE, Metzgeroth G, Hastka J (2021). The evaluation of iron deficiency and iron overload. Dtsch Arztebl Int.

[14] Nashwan AJ, Yassin MA, Mohamed Ibrahim MI, Abdul Rahim HF, Shraim M (2022). Iron overload in chronic kidney disease: Less ferritin, more T2* MRI. Front Med.

[15] Coyne DW (2017). Iron overload in dialysis patients: Rust or bust?. Kidney Int Rep.

[16] Nashwan AJ, Yassin MA (2023). Deferasirox in patients with chronic kidney disease: Assessing the potential benefits and challenges. J Blood Med.

[17] Nashwan AJ, Yassin MA, Abd-Alrazaq A, Shuweihdi F, Othman M, Abdul Rahim HF, Shraim M (2023). Hepatic and cardiac iron overload quantified by magnetic resonance imaging in patients on hemodialysis: A systematic review and meta-analysis. Hemodial Int.

[18] Macdougall IC, Bock AH, Carrera F (2014). FIND-CKD: A randomized trial of intravenous ferric carboxymaltose versus oral iron in patients with chronic kidney disease and iron deficiency anaemia. Nephrol Dial Transplant.

[19] Fishbane S, Mathew A, Vaziri ND (2014). Iron toxicity: Relevance for dialysis patients. Nephrol Dial Transplant.

[20] Macdougall IC, White C, Anker SD (2019). Intravenous iron in patients undergoing maintenance hemodialysis. N Engl J Med.

[21] Fujisawa H, Nakayama M, Haruyama N (2023). Association between iron status markers and kidney outcome in patients with chronic kidney disease. Sci Rep.

[22] Yu PH, Chao YL, Kuo IC, Niu SW, Chiu YW, Chang JM, Hung CC (2023). The association between iron deficiency and renal outcomes is modified by sex and anemia in patients with chronic kidney disease stage 1-4. J Pers Med.

[23] Gafter-Gvili A, Schechter A, Rozen-Zvi B (2019). Iron deficiency anemia in chronic kidney disease. Acta Haematol.

[24] Sathian B, Asim M, Banerjee I (2020). Impact of COVID-19 on clinical trials and clinical research: A systematic review. Nepal J Epidemiol.

